# Prognostic values of inflammatory indexes in bevacizumab-treated patients with advanced non-small-cell lung cancer

**DOI:** 10.2144/fsoa-2021-0162

**Published:** 2022-04-14

**Authors:** Jingru Yang, Mingliang Deng, Minghong Bi, Yaping Wang, Xuxu Qiao, Shanshan Zhang

**Affiliations:** 1 Department of Medical Oncology, The First Affiliated Hospital of Bengbu Medical College, Bengbu, Anhui Province, China; 2 Department of Neurosurgery, The First Affiliated Hospital of Bengbu Medical College, Bengbu, Anhui Province, China

**Keywords:** bevacizumab, lymphocyte-to-monocyte ratio, neutrophil-to-lymphocyte ratio, non-small-cell lung cancer, platelet-to-lymphocyte ratio, systemic immune-inflammation index

## Abstract

**Purpose:**

Inflammatory indexes, including neutrophil-to-lymphocyte ratio (NLR), platelet-to-lymphocyte ratio (PLR), systemic immune-inflammation index (SII) and lymphocyte-to-monocyte ratio (LMR), have been confirmed as prognostic factors in multiple manigances. However, the prognostic value of these parameters in bevacizumab-treated non-small-cell lung cancer (NSCLC) is still not clear.

**Methods:**

We retrospectively studied 119 patients with advanced NSCLC who received bevacizumab treatment. The associations of pretreatment NLR, PLR, SII and LMR with progression-free survival (PFS) and overall survival (OS) were analyzed.

**Results & Conclusion:**

The median PFS and OS of patients with high baseline NLR, PLR and SII and low LMR were significantly decreased than those of patients with low baseline NLR, PLR and SII and high LMR. Multivariable analysis indicated that high baseline SII was independently related with inferior prognosis, and baseline LMR was an independent predictor for OS.

## Plain language summary

In this study we retrospectively studied 119 patients with advanced non-small-cell lung cancer receiving bevacizumab treatment. We found that the prognosis of the patients with high baseline neutrophil-to-lymphocyte ratio (NLR), platelet-to-lymphocyte ratio (PLR) and systemic immune-inflammation index (SII) and low lymphocyte-to-monocyte ratio (LMR) were significantly poorer than those in patients with low baseline NLR, PLR and SII and high LMR. Multivariable analysis indicated that high baseline SII was independently related with inferior progression-free survival and overall survival, and that baseline LMR was an independent predictor for overall survival. This study suggests that we can predict the efficacy of bevacizumab by analyzing several blood cell count indexes.

Lung cancer ranks first both in the incidence and mortality rate among malignant tumors worldwide [1]. Bevacizumab is a monoclonal antibody which exerts its anti-angiogenesis ability by targeting VEGF. At present, bevacizumab combined with platinum-based doublet chemotherapy has been widely administered as one of the standard first-line therapeutic options for patients with metastatic non-small-cell lung cancer (NSCLC) [2]. However, there are still no validated clinical or biological biomarkers to predict the effects of and resistance to bevacizumab.

Inflammatory cells, including lymphocytes, neutrophils and monocytes, are a key component of the tumor microenvironment and substantially contribute to tumor proliferation, angiogenesis and metastasis [3]. Several inflammatory indexes, such as neutrophil-to-lymphocyte ratio (NLR), platelet-to-lymphocyte ratio (PLR), systemic immune-inflammation index (SII) and lymphocyte-to-monocyte ratio (LMR), have been increasingly recognized as prognostic hematological biomarkers in a variety of malignancies, including colorectal cancer, ovarian cancer, prostate cancer, hepatocellular carcinoma and NSCLC [4–9].

We performed this retrospective study with the purpose of assessing the prognostic value of NLR, PLR, SII and LMR in advanced NSCLC patients receiving bevacizumab and chemotherapy as the first-line treatment. If validated, these reproducible, cost effective and easily available peripheral immune-related biomarkers could be particularly useful in identifying NSCLC patients who may benefit from bevacizumab treatment.

## Patients & methods

### Patients

A total of 119 patients diagnosed with advanced NSCLC (stage IIIB/IV) at the First Affiliated Hospital of Bengbu Medical College between January 2017 and April 2021 were enrolled in our retrospective study. Eligible patients received at least one infusion of bevacizumab at a dose of 15 mg/kg together with paclitaxel (175 mg/m^2^) and carboplatin (area under the curve = 5) every 21 days. The therapy was continued until tumor progression, intolerable side effects, withdrawal or death. Patients who had used steroids or immunomodulatory drugs 1 month before the initiation of bevacizumab, or had coexisting active inflammatory diseases or immunodeficiencies, were excluded.

The patients’ clinical records were collected, including laboratory complete blood counts with differential count at the initiation of bevacizumab administration, sex, age, smoking history, *EGFR* mutation status, Eastern Co-operative Oncology Group performance status (ECOG PS) and the occurrence of bone, liver or brain metastases. All data were retrospectively collected by using the electronic patient medical records system to ensure consistent data collection. Disease progression was identified based on the Response Evaluation Criteria in Solid Tumors version 1.1. Progression-free survival (PFS) was calculated as the interval elapsed from the initiation of bevacizumab to first disease progression or death for any reason. Overall survival (OS) was characterized by the interval from commencing bevacizumab treatment to death or last follow-up. The follow-up of all patients ended on 15 November 2021.

### Statistical analysis

Descriptive analysis was applied for all variables. Categorical parameters were described as frequencies and percentages, while continuous parameters were described as medians with ranges.

NLR was defined as the ratio of absolute neutrophil number to absolute lymphocyte number; PLR was derived from the quotient of the absolute platelet and lymphocyte count; SII was defined as the formula PLR × neutrophil count; and LMR was defined as the quotient of the absolute lymphocyte and monocyte count. The baseline markers were defined as the baseline counts immediately before the first cycle of bevacizumab.

**Figure 1.**
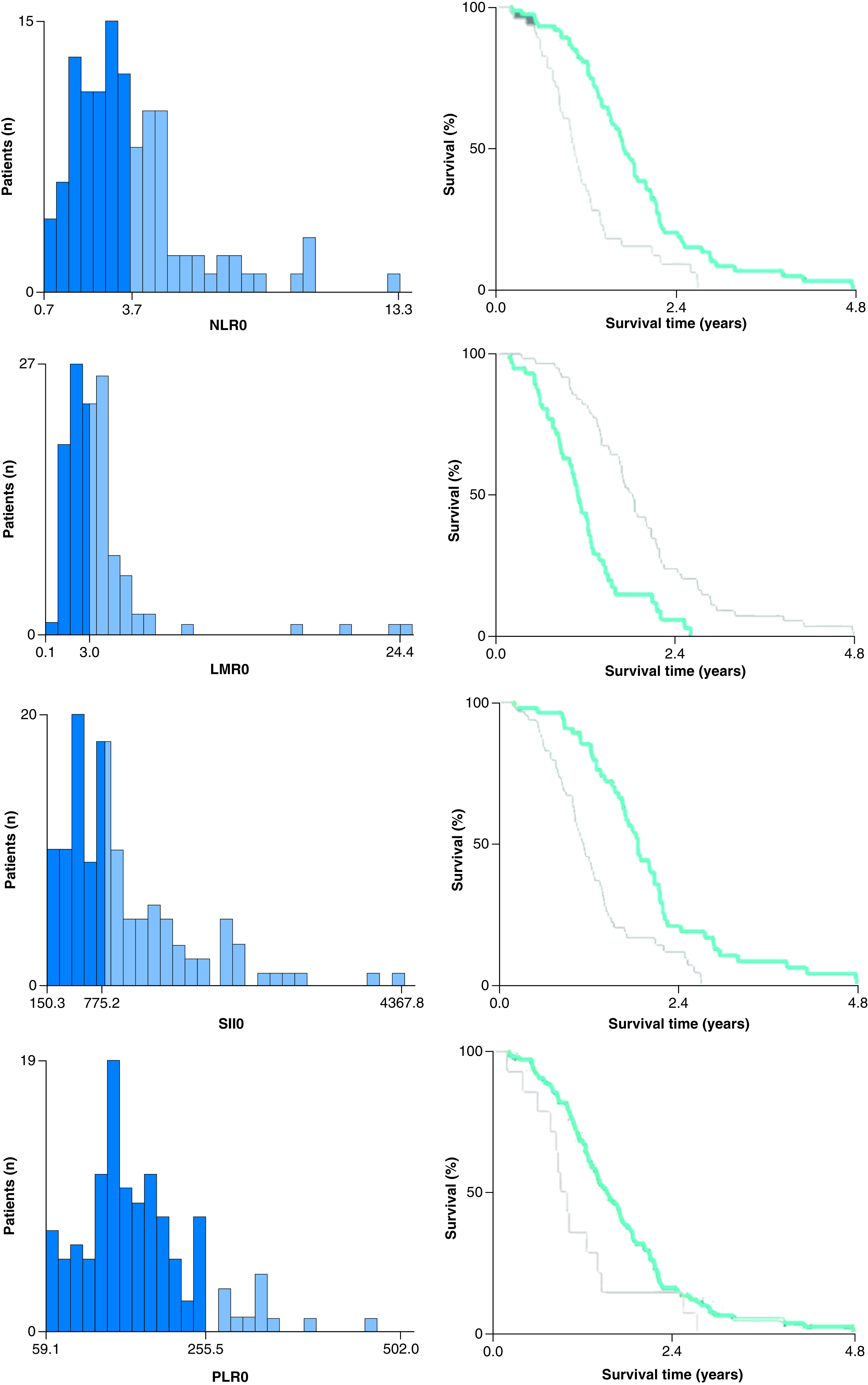
X-tile analysis of overall survival based on neutrophil-to-lymphocyte ratio, lymphocyte-to-monocyte ratio, systemic immune-inflammation index and platelet-to-lymphocyte ratio. NLR: Neutrophil-to-lymphocyte ratio; LMR: Lymphocyte-to-monocyte ratio; PLR: Platelet-to-lymphocyte ratio; SII: Systemic immune-inflammation index.

The best cutoffs influencing the prognosis of baseline NLR/PLR/SII/LMR were defined using the X-tile v.3.6.1 software and the minimum p-value method as follows: 3.7 for NLR, 255.5 for PLR, 775.2 for SII and 3 for LMR, respectively (Figure 1) [10]. Patients were categorized into a high baseline NLR/SII/LMR/PLR group and a low baseline NLR/SII/LMR/PLR group according to the cutoff values.

The relationship between the baseline NLR, SII, LMR and PLR levels and the patients’ clinical characteristics was calculated using the chi-square test. The distributions of PFS and OS were assessed using the Kaplan–Meier method, and the log-rank test was utilized for comparison. Univariate and multivariate Cox proportional hazards regression models were applied to evaluate the contribution of each potential prognostic factor to survival. Parameters entered into the multivariate analysis were chosen on the basis of the clinical relevance and statistical significance suggested by univariate Cox regression analysis (p < 0.05). A two-tailed p-value < 0.05 was regarded as statistically significant. All data were analyzed using the SPSS v. 24.0 software (IBM Corp., NY, USA).

## Results

### Characteristics of patients

A total of 119 patients with advanced NSCLC who received bevacizumab and chemotherapy as first-line treatment from January 2017 to April 2021 were included in the research. Baseline characteristics of all included patients are shown in Table 1. The median age was 61 years at diagnosis (range: 36–80 years). Sixty-seven patients (56.3%) were men and 73 (61.3%) were smokers or had a previous history of smoking. A majority of patients (72%) had an ECOG PS of 1 or 2. In relation to tumor characteristics, the most common site of metastasis was bone (43.7%), followed by brain (29.4%) and liver (15.1%). Regarding *EGFR* mutation status, 36.1% had a mutation, while 63.9% had the wild-type gene.

**Table 1.** Characteristics of patients.

Characteristics	n (%), total = 119
Age (years)	
Median (range)	61 (36–80)
<60	55 (46.2)
≥60	64 (53.8)
Gender	
Female	52 (43.7)
Male	67 (56.3)
Smoking status	
Yes	73 (61.3)
No	46 (38.7)
ECOG PS	
0	33 (28)
1–2	86 (72)
*EGFR* status	
Mutation	43 (36.1)
Negative	76 (63.9)
Metastatic sites	
Brain	35 (29.4)
Bone	52 (43.7)
Liver	18 (15.1)

### Correlation between inflammatory indexes & clinical characteristics

Before the initiation of bevacizumab treatment, a total of 73 (61.3%) patients had low NLR and 105 (88.2%) low PLR, while 56 (47.1%) and 65(54.6%) patients had low SII and high LMR, respectively. We compared the baseline characteristics of patients grouped by inflammatory indexes cut-offs (Tables 2 and 3). Male patients trend to be more frequent in the high NLR (≥3.7), SII (≥755.2) and LMR (≥3) group. Patients with low NLR (<3.7) or low LMR (<3) exhibited more frequently an ECOG PS of 1–2. No significant difference was found among different groups with respect to any other characteristics.

**Table 2.** Patients' baseline characteristics classified by neutrophil-to-lymphocyte ratio and platelet-to-lymphocyte ratio (n = 119).

	NLR			PLR		
	<3.7, n (%)	≥3.7, n (%)	p-value	<255.5, n (%)	≥255.5, n (%)	p-value
Age (years)						
<60	35 (47.9)	20 (43.5)	0.71	48 (45.7)	7 (50.0)	0.78
≥60	38 (52.1)	26 (56.5)		57 (54.3)	7 (50.0)	
Gender						
Female	38 (52.1)	14 (30.4)	0.02	45 (42.9)	7 (50.0)	0.78
Male	35 (47.9)	32 (69.6)		60 (57.1)	7 (50.0)	
Smoking status						
No	27 (37)	18 (39.1)	0.8	38 (36.2)	7 (50.0)	0.38
Yes	46 (63)	28 (60.9)		67 (63.8)	7 (50.0)	
ECOG PS						
0	15 (20.5)	18 (39.1)	0.03	27 (25.7)	6 (42.9)	0.21
≥1	58 (79.5)	28 (60.9)		78 (74.3)	8 (57.1)	
*EGFR* status						
Wild-type	43 (58.9)	35 (76.1)	0.07	70 (66.7)	8 (57.1)	0.55
Mutation	30 (41.1)	11 (23.9)		35 (33.3)	6 (42.9)	
Brain metastasis						
No	51 (69.6)	33 (71.7)	1	74 (70.5)	10 (71.4)	1
Yes	22 (30.1)	13 (28.3)		31 (29.5)	4 (28.6)	
Bone metastasis						
No	36 (49.3)	31 (67.4)	0.06	56 (53.3)	11 (78.6)	0.09
Yes	37 (50.7)	15 (32.6)		49 (46.7)	3 (21.4)	
Liver metastasis						
No	61 (83.6)	39 (84.8)	1	88 (83.8)	12 (85.7)	1
Yes	12 (16.4)	7 (15.2)		17 (16.2)	2 (14.3)	

ECOG PS: Eastern Co-operative Oncology Group performance status; NLR: Neutrophil-to-lymphocyte ratio; PLR: Platelet-to-lymphocyte ratio.

**Table 3.** Patients' baseline characteristics classified by systemic immune-inflammation index and lymphocyte-to-monocyte ratio (n = 119).

	SII			LMR		
	<755.2, n (%)	≥755.2, n (%)	p-value	<3, n (%)	≥3, n (%)	p-value
Age (years)						
<60	28 (50.0)	27 (42.9)	0.47	36 (55.4)	19 (35.2)	0.04
≥60	28 (50.0)	36 (57.1)		29 (44.6)	35 (64.8)	
Gender						
Female	33 (58.9)	19 (30.2)	0.002	41 (63.1)	11 (20.4)	<0.01
Male	23 (41.1)	44 (69.8)		24 (36.9)	43 (79.6)	
Smoking status						
No	21 (37.5)	24 (38.1)	1	27 (41.5)	18 (33.3)	0.45
Yes	35 (62.5)	39 (61.9)		38 (58.5)	36 (66.7)	
ECOG PS						
0	13 (23.2)	20 (31.7)	0.31	13 (20.0)	20 (37.0)	0.04
≥1	43 (76.8)	43 (68.3)		52 (80.0)	34 (63.0)	
*EGFR* status						
Wild-type	33 (58.9)	45 (71.4)	0.18	41 (63.1)	37 (68.5)	0.57
Mutation	23 (41.1)	18 (28.6)		24 (36.9)	17 (31.5)	
Brain metastasis						
No	40 (71.4)	44 (69.8)	1	45 (69.2)	39 (72.2)	0.84
Yes	16 (28.6)	19 (30.2)		20 (30.8)	15 (27.8)	
Bone metastasis						
No	27 (48.2)	40 (63.5)	0.1	31 (47.7)	36 (66.7)	0.04
Yes	29 (51.8)	23 (36.5)		34 (52.3)	18 (33.3)	
Liver metastasis						
No	48 (85.7)	52 (82.5)	0.8	57 (87.7)	43 (79.6)	0.32
Yes	8 (14.3)	11 (17.5)		8 (12.3)	11 (20.4)	

ECOG PS: Eastern Co-operative Oncology Group performance status; LMR: Lymphocyte-to-monocyte ratio; SII: Systemic immune-inflammation index.

### Correlation between inflammatory indexes & survival outcomes

**Figure 2.**
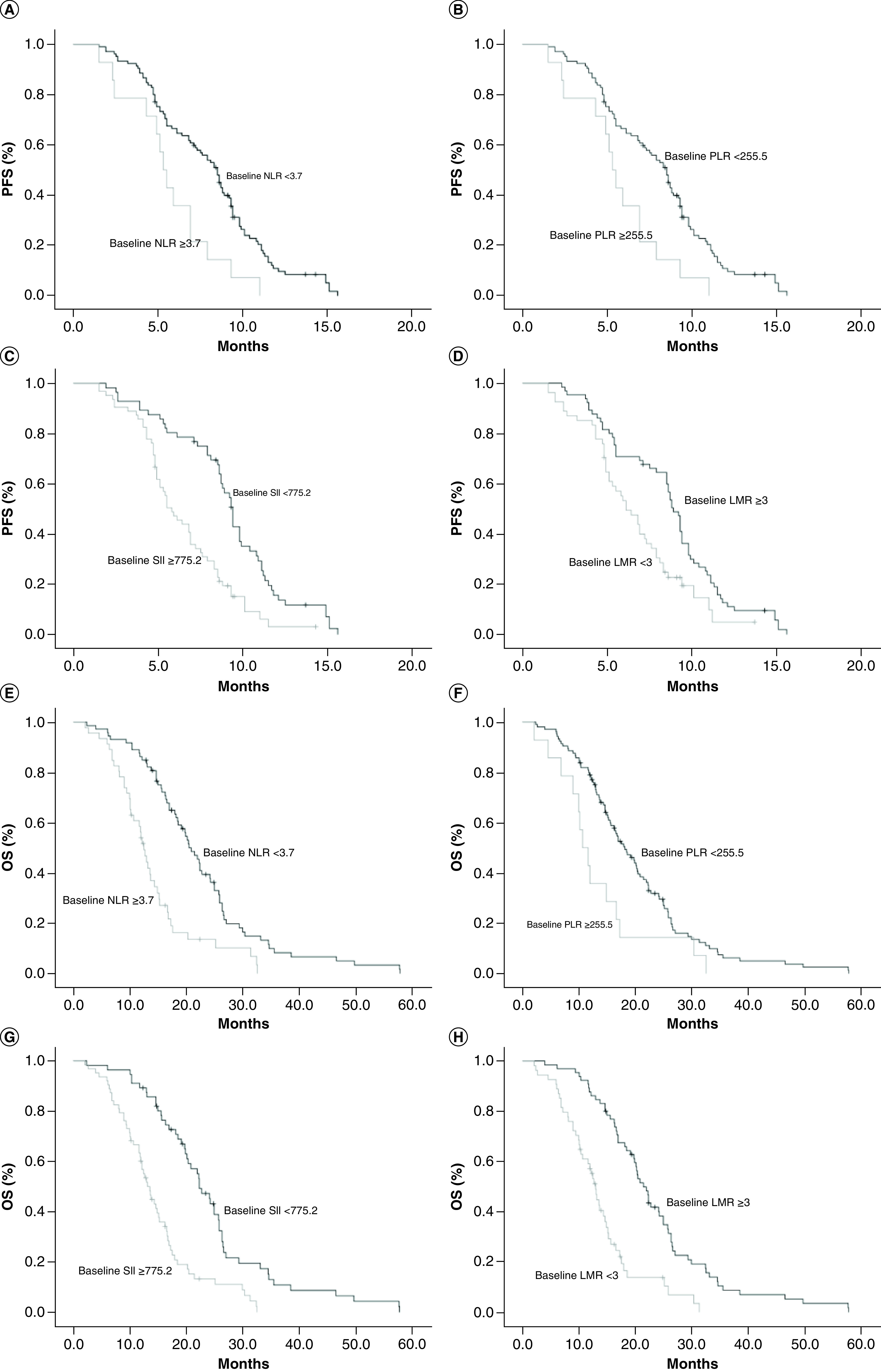
Kaplan–Meier curves comparing progression-free survival and overall survival according to neutrophil-to-lymphocyte ratio, platelet-to-lymphocyte ratio, systemic immune-inflammation index and lymphocyte-to-monocyte ratio. **(A)** PFS–NLR. **(B)** PFS–PLR. **(C)** PFS–SII. **(D)** PFS–LMR. **(E)** OS–NLR. **(F)** OS–PLR. **(G)** OS–SII. **(H)** OS–LMR. LMR: Lymphocyte-to-monocyte ratio; NLR: Neutrophil-to-lymphocyte ratio; OS: Overall survival; PFS: Progression-free survival; PLR: Platelet-to-lymphocyte ratio; SII: Systemic immune-inflammation index.

The median PFS and OS for the entire cohort included in this study were 7.9 months (95% CI: 6.8–8.6) and 16.2 months (95% CI: 14.6–17.9), respectively. Kaplan–Meier survival analysis suggested that patients with high baseline NLR (≥3.7), PLR (≥255.5) and SII (≥775.2) and low LMR (<3) showed significantly worse PFS than those with low baseline NLR (<3.7), PLR (<255.5) and SII (<775.2) and high LMR (≥3), with median PFS of 5.9 versus 8.8 months (p = 0.002; Figure 2A), 5.3 versus 8.5 months (p = 0.004; Figure 2B), 5.8 versus 9.4 months (p < 0.001; Figure 2C) and 6.1 versus 8.9 months (p = 0.003; Figure 2D), respectively.

We also compared the relationship of OS with the inflammatory indexes. Figure 2E–H shows that the OS in patients with baseline NLR ≥3.7, PLR ≥255.5, SII ≥775.2 and LMR <3 were significantly inferior to OS in those with baseline NLR <3.7, PLR <775.2, SII <775.2 and LMR ≥3, with median OS of 12.4 versus 20.4 months (p < 0.001; Figure 2E), 10.6 versus 18.1 months (p = 0.018; Figure 2F), 13.1 versus 22.3 months (p < 0.001; Figure 2G) and 12.9 versus 21.4 months (p < 0.001; Figure 2H), respectively.

### Univariate & multivariate analysis for PFS & OS

Univariate and multivariate Cox regression were performed to analyze the prognostic importance of patients’ baseline characteristics and inflammatory indexes, and the results are summarized in Tables 4 & 5.

**Table 4.** Univariate and multivariable analysis of variables correlated to progression-free survival.

	Univariable model			Multivariable model		
	HR	95% CI	p-value	HR	95% CI	p-value
Age (≥60 vs <60)	1.172	0.800–1.716	0.415			
Gender (male vs female)	1.736	1.167–2.584	0.007	–	–	0.119
Smoking status (ever vs never)	1.254	0.845–1.861	0.261			
ECOG PS (≥1 vs <1)	1.083	0.707–1.660	0.714			
*EGFR* mutation (yes vs no)	0.843	0.566–1.256	0.401			
Bone metastasis (yes vs no)	0.915	0.624–1.342	0.649			
Brain metastasis (yes vs no)	1.150	0.759–1.742	0.509			
Liver metastasis (yes vs no)	2.226	1.321–3.752	0.003	2.080	1.232–3.509	0.006
Baseline NLR (≥3.7 vs <3.7)	1.881	1.251–2.827	0.002	–	–	0.767
Baseline PLR (≥255.5 vs <255.5)	2.274	1.279–4.041	0.005	–	–	0.181
Baseline SII (≥775.2 vs <775.2)	2.407	1.061–3.619	<0.001	2.338	1.554–3.518	<0.001
Baseline LMR (<3 vs ≥3)	1.831	1.220–2.749	0.004	–	–	0.378

ECOG PS: Eastern Co-operative Oncology Group performance status; HR: Hazard ratio; LMR: Lymphocyte-to-monocyte ratio; NLR: Neutrophil-to-lymphocyte ratio; PLR: Platelet-to-lymphocyte ratio; SII: Systemic immune-inflammation index.

**Table 5.** Univariate and multivariable analysis of variables correlated to overall survival.

	Univariable model			Multivariable model		
	HR	95% CI	p-value	HR	95% CI	p-value
Age (≥60 vs <60)	1.107	0.755–1.622	0.604			
Gender (male vs female)	1.948	1.308–2.902	0.001	–	–	0.280
Smoking status (ever vs never)	1.556	1.032–2.349	0.035			
ECOG PS (≥1 vs <1)	1.028	0.670–1.577	0.901			
*EGFR* mutation (yes vs no)	0.414	0.265–0.647	<0.001	0.385	0.234–0.609	<0.001
Bone metastasis (yes vs no)	0.926	0.630–1.362	0.696			
Brain metastasis (yes vs no)	1.746	1.140–2.675	0.010	1.582	1.027–2.436	0.037
Liver metastasis (yes vs no)	1.756	1.062–2.904	0.028	–	–	0.357
Baseline NLR (≥3.7 vs <3.7)	2.584	1.709–3.908	<0.001	–	–	0.711
Baseline PLR (≥255.5 vs <255.5)	1.965	1.111–3.474	0.020	–	–	0.662
Baseline SII (≥775.2 vs <775.2)	2.799	1.850–4.233	<0.001	1.846	1.165–2.923	0.009
Baseline LMR (<3 vs ≥3)	2.998	1.969–4.565	<0.001	2.619	1.631–4.205	<0.001

ECOG PS: Eastern Co-operative Oncology Group performance status; HR: Hazard ratio; LMR: Lymphocyte-to-monocyte ratio; NLR: Neutrophil-to-lymphocyte ratio; PLR: Platelet-to-lymphocyte ratio; SII: Systemic immune-inflammation index.

In univariate analysis of PFS, no significant associations with PFS were found for patients’ age, smoking history, ECOG PS score, EGFR status, or bone or brain metastasis. Patients with baseline NLR ≥3.7 (hazard ratio [HR]: 1.881; 95% CI: 1.251–2.82; p = 0.002), baseline PLR ≥255.5 (HR: 2.274; 95% CI: 1.279–4.041; p = 0.005), baseline SII ≥775.2 (HR: 2.407; 95% CI: 1.061–3.619; p < 0.001) and baseline LMR <3 (HR: 1.831; 95% CI: 1.220–2.749; p = 0.004) experienced shorter PFS. Furthermore, multivariate analysis demonstrated that only baseline SII ≥775.2 (HR: 2.338; 95% CI: 1.554–3.518; p < 0.001) and occurrence of liver metastasis (HR: 2.080; 95% CI: 1.232–3.509; p = 0.006) remained to be independently correlated with inferior PFS (Table 4).

In univariate analysis of OS, the presence of brain metastasis (HR: 1.746; 95% CI: 1.140–2.675; p = 0.010), liver metastasis (HR: 1.756; 95% CI: 1.062–2.904; p = 0.028), baseline NLR ≥3.7 (HR: 2.584; 95% CI: 1.709–3.908; p < 0.001), baseline PLR ≥255.5 (HR: 1.965; 95% CI: 1.111–3.474; p = 0.020), baseline SII ≥775.2 (HR: 2.799; 95% CI: 1.850–4.233; p < 0.001) and baseline LMR <4 (HR: 2.998; 95% CI: 1.969–4.565; p < 0.001) were indicated to be unfavorable prognostic factors. *EGFR* mutation (HR: 0.414; 95% CI: 0.265–0.647; p < 0.001) was indicated to be a favorable prognostic factor. The prognostic value of age, smoking status, ECOG PS and bone metastasis were evaluated and showed no significant correlation with OS. Furthermore, multivariate Cox regression analysis showed that baseline LMR ≥3 (HR: 2.619; 95% CI: 1.631–4.205; p < 0.001), baseline SII <775.2 (HR: 1.846; 95% CI: 1.165–2.923; p = 0.009), together with mutant *EGFR* (HR: 0.385; 95% CI 0.243–0.609; p < 0.001) and no brain metastasis (HR: 1.582; 95% CI 1.027–2.436; p = 0.037) remained strongly associated with superior OS (Table 5).

## Discussion

The present study suggested that in advanced NSCLC patients receiving bevacizumab plus chemotherapy as first-line treatment, the baseline systemic inflammatory indexes exerted an influence on survival. In our cohort we observed that NLR ≥3.7, PLR ≥255.5, SII ≥775.2 and LMR <3 at baseline were significantly related to diminished PFS and OS. In multivariate analysis, high baseline SII was suggested to be an independent prognostic marker of PFS and OS, and low baseline LMR was found to contribute significantly to the prediction of OS. However, baseline NLR and baseline PLR lost their predictive values in terms of PFS and OS. Furthermore, the multivariate analysis also suggested the association of superior efficacy of bevacizumab in patients with *EGFR* gene mutation.

The underlying mechanism associated with the inflammatory indexes NLR, SII and LMR and prognosis could be partly explained by the association of cancer-related inflammation with tumor promotion and progression. Inflammation is regarded as the seventh hallmark of tumors and plays a major role in the proliferation, invasion and metastasis of multiple malignancies. Fewer lymphocytes and more neutrophils and monocytes are associated with progression or poor prognosis in various tumors [3,11,12]. Mounting evidence suggests that VEGF is not only produced by tumor cells, but is also released to the tumor microenvironment by platelets and inflammatory cells such as neutrophils and monocytes during the tumor-associated inflammatory response and hypoxia [13]. VEGF is involved in fostering immunosuppression in the tumor by downregulating the proliferation and differentiation of antigen-presenting cells as well as effector T cells while activating immunosuppressive cells including myeloid-derived suppressor cells, tumor-associated macrophages (TAMs) and regulatory T cells [14,15]. The peritumoral infiltration of these suppressive immune cells not only releases multiple angiostimulatory growth factors, but also facilitates and stimulates alternative angiogenesis pathways which contribute to the resistance to bevacizumab [16]. In addition, the resistance to bevacizumab will aggravate the anaerobic state in the tumor microenvironment and enhance recruitment of neutrophils and macrophages, which can establish a positive feedback that further impairs the immune response and promotes tumor growth [17–19].

Neutrophils, derived from the peripheral blood, are recruited at the site of inflammation and the tumor, and their number is always increased in tumors compared with healthy tissues [20]. Neutrophilia is able to abolish the function of immune cells, increase peritumoral aggregation of macrophages, increase C-reactive protein (CRP) and reduce albumin synthesis. In addition, neutrophils contribute to tumor angiogenesis by delivering angiogenic factors including VEGF, protease, IL-1, IL-6 and IL-8, and eventually resulting in tumor progression [21]. Early studies confirmed that lymphocytes, especially tumor-infiltrating lymphocytes, had a major impact on the antitumor inflammatory response by inducing cytotoxic cell apoptosis and suppressing the proliferation and dissemination of tumor cells; thus increased numbers of tumor-infiltrating lymphocytes are correlated with better prognosis [22]. Lymphocytopenia is frequently found in malignancies, indicating an immune resistance status [23]. This might be due to the higher susceptibility of T lymphocytes to apoptosis, caused by a chronic activation state in solid tumor tissues [24]. Peripheral monocytes accumulate into the tumor tissue and are differentiated into TAMs under the action of different tumor-derived chemokines, such as VEGFA and CCL-2; TAMs are able to divide into tumor-supportive M2-polarized macrophages, which promote angiogenesis by secreting different proangiogenic factors, including VEGFA, MMP-9, bFGF and ADM [25,26].

Decreased lymphocytes and increased neutrophils, platelets and monocytes, in combination with higher NLR, PLR and SII and lower LMR, are confirmed to be helpful for estimating the status of tumor immunity during bevacizumab treatment, and have a predictive role in various malignancies [27–30]. Our research is consistent with previous research suggesting that high NLR, PLR and SII and low LMR are independent factors predicting inferior survival in several malignancies, including lung, colorectal, ovarian and gastric cancer [7,31–33]. Especially in lung cancer, these inflammatory indexes have shown an association with poor survival outcomes in small-cell lung cancer and early and advanced NSCLC patients, and were able to predict the resistance to chemotherapy and target therapy as well as immunotherapy. In a comprehensive retrospective study, Mandaliya *et al.* illustrated that in patients with stage IV NSCLC, high baseline NLR and PLR and low baseline LMR, the advanced lung cancer inflammation index had a significant association with poorer survival [32]. In another retrospective study, NLR was demonstrated to be a useful prognostic index for first-line chemotherapy in stage IIIB or IV NSCLC patients [34]. Furthermore, Chan *et al.* showed that in a subpopulation of *EGFR*-mutated NSCLC patients, baseline NLR was recognized as an independent prognostic factor across early- and late-stage disease [35]. In addition, Cao *et al.* suggested an association of high pretreatment NLR with worse outcome in NSCLC patients treated with nivolumab [36]. Botta *et al.* found that NLR could be a useful prognostic marker for NSCLC patients treated with bevacizumab [4]. Li *et al.* also confirmed that decreased NLR, PLR and SII and elevated LMR could indicate a favorable response to bevacizumab in NSCLC patients [37].

Nevertheless, our present study had several limitations. First, the peripheral blood cell parameters such as neutrophils, platelets and lymphocytes are nonspecific parameters and may be influenced by some confounding factors including infections, the use of steroid drugs or cancer-related complications. We excluded patients with active infections or autoimmune disease from this study, but we could not confirm whether latent infections existed. Second, the low infiltration of CD4^+^ T lymphocytes and CD8^+^ T lymphocytes in the tumor microenvironment has been found to predict poorer survival in various malignancies. Compared with CD4^+^ helper T cells, the infiltration of CD8^+^ T cells in tumor was relatively higher [38]. Nevertheless, we could not further analyze the specific T-lymphocyte subgroups due to the retrospective nature of the study. Thirdly, the possibility of selection bias and confounders could not be completely avoided because of the inherent characteristics of retrospective studies.

## Conclusion

In conclusion, our study demonstrated that high baseline NLR, PLR and SII and low baseline LMR were significantly associated with inferior PFS and OS, and that pretreatment LMR and SII appeared to be independent prognostic indexes for advanced NSCLC patients treated with first-line bevacizumab plus chemotherapy. An increasing body of evidence has proved the important role of systemic inflammatory factors in tumor aggressiveness. Investigation of a further larger prospective dataset is warranted to validate our findings.

**Summary points**

- This study was designed to investigate the role of several inflammatory indexes in predicting the efficacy of bevacizumab in patients with advanced non-small-cell lung cancer (NSCLC).
- In this retrospective study we aimed to evaluated the relationship between neutrophil-to-lymphocyte ratio (NLR), platelet-to-lymphocyte ratio (PLR), systemic immune-inflammation index (SII) and lymphocyte-to-monocyte ratio (LMR) and the prognosis of 119 patients with advanced NSCLC treated with bevacizumab combined with platinum-based doublet chemotherapy. Statistical analysis was evaluated using the Kaplan–Meier method and Cox regression.
- The study suggested that median progression-free survival (PFS) and overall survival (OS) of patients with high baseline NLR (≥3.7), PLR (≥255.5) and SII (≥775.2) and low baseline LMR (<3) were significantly shorter than those of patients with low baseline NLR (<3.7), PLR (<255.5) and SII (<775.2) and high LMR (≥3).
- Multivariable analysis indicated that high baseline SII was independently related to inferior PFS and OS, and that baseline LMR was an independent predictor for OS.

## References

[1] Bade BC, Dela Cruz CS. Lung cancer 2020: epidemiology, etiology, and prevention. Clin. Chest Med. 41(1), 1–24 (2020). 32008623 10.1016/j.ccm.2019.10.001

[2] Hanna N, Johnson D, Temin S Systemic therapy for stage IV non-small-cell lung cancer: American Society of Clinical Oncology Clinical Practice Guideline Update. J. Clin. Oncol. 35(30), 3484–3515 (2017). 28806116 10.1200/JCO.2017.74.6065

[3] Diakos CI, Charles KA, McMillan DC, Clarke SJ. Cancer-related inflammation and treatment effectiveness. Lancet Oncol. 15(11), e493–e503 (2014). 25281468 10.1016/S1470-2045(14)70263-3

[4] Botta C, Barbieri V, Ciliberto D Systemic inflammatory status at baseline predicts bevacizumab benefit in advanced non-small cell lung cancer patients. Cancer Biol. Ther. 14(6), 469–475 (2013). 23760488 10.4161/cbt.24425 PMC3813562

[5] Guthrie GJ, Charles KA, Roxburgh CS, Horgan PG, McMillan DC, Clarke SJ. The systemic inflammation-based neutrophil-lymphocyte ratio: experience in patients with cancer. Crit. Rev. Oncol. Hematol. 88(1), 218–230 (2013). 23602134 10.1016/j.critrevonc.2013.03.010

[6] Rossi S, Basso M, Strippoli A Are markers of systemic inflammation good prognostic indicators in colorectal cancer? Clin. Colorectal Cancer 16(4), 264–274 (2017). 28412137 10.1016/j.clcc.2017.03.015

[7] Farolfi A, Scarpi E, Greco F Inflammatory indexes as predictive factors for platinum sensitivity and as prognostic factors in recurrent epithelial ovarian cancer patients: a MITO24 retrospective study. Sci. Rep. 10(1), 18190 (2020). 33097745 10.1038/s41598-020-75316-x PMC7585431

[8] Templeton AJ, Pezaro C, Omlin A Simple prognostic score for metastatic castration-resistant prostate cancer with incorporation of neutrophil-to-lymphocyte ratio. Cancer 120(21), 3346–3352 (2014). 24995769 10.1002/cncr.28890

[9] Yang Z, Zhang J, Lu Y Aspartate aminotransferase–lymphocyte ratio index and systemic immune-inflammation index predict overall survival in HBV-related hepatocellular carcinoma patients after transcatheter arterial chemoembolization. Oncotarget 6(40), 43090–43098 (2015). 26506519 10.18632/oncotarget.5719 PMC4767493

[10] Camp RL, Dolled-Filhart M, Rimm DL.X-tile: a new bio-informatics tool for biomarker assessment and outcome-based cut-point optimization. Clin Cancer Res. 10(21), 7252–7259 (2004). 15534099 10.1158/1078-0432.CCR-04-0713

[11] Greten FR, Grivennikov SI. Inflammation and cancer: triggers, mechanisms, and consequences. Immunity 51(1), 27–41 (2019). 31315034 10.1016/j.immuni.2019.06.025 PMC6831096

[12] Kumar R, Geuna E, Michalarea V The neutrophil-lymphocyte ratio and its utilisation for the management of cancer patients in early clinical trials. Br. J. Cancer 112(7), 1157–1165 (2015). 25719834 10.1038/bjc.2015.67 PMC4385959

[13] Koehne P, Willam C, Strauss E, Schindler R, Eckardt KU, Buhrer C. Lack of hypoxic stimulation of VEGF secretion from neutrophils and platelets. Am. J. Physiol. Heart Circ. Physiol. 279(2), H817–H824 (2000). 10924082 10.1152/ajpheart.2000.279.2.H817

[14] Rahma OE, Hodi FS. The intersection between tumor angiogenesis and immune suppression. Clin. Cancer Res. 25(18), 5449–5457 (2019). 30944124 10.1158/1078-0432.CCR-18-1543

[15] Ramjiawan RR, Griffioen AW, Duda DG. Anti-angiogenesis for cancer revisited: is there a role for combinations with immunotherapy? Angiogenesis 20(2), 185–204 (2017). 28361267 10.1007/s10456-017-9552-y PMC5439974

[16] Huijbers EJ, van Beijnum JR, Thijssen VL, Sabrkhany S, Nowak-Sliwinska P, Griffioen AW. Role of the tumor stroma in resistance to anti-angiogenic therapy. Drug Resist. Updat. 25, 26–37 (2016). 27155374 10.1016/j.drup.2016.02.002

[17] Brandau S, Moses K, Lang S. The kinship of neutrophils and granulocytic myeloid-derived suppressor cells in cancer: cousins, siblings or twins? Semin. Cancer Biol. 23(3), 171–182 (2013). 23459190 10.1016/j.semcancer.2013.02.007

[18] Lu T, Ramakrishnan R, Altiok S Tumor-infiltrating myeloid cells induce tumor cell resistance to cytotoxic T cells in mice. J. Clin. Invest. 121(10), 4015–4029 (2011). 21911941 10.1172/JCI45862 PMC3195459

[19] Bottsford-Miller JN, Coleman RL, Sood AK. Resistance and escape from antiangiogenesis therapy: clinical implications and future strategies. J. Clin. Oncol. 30(32), 4026–4034 (2012). 23008289 10.1200/JCO.2012.41.9242 PMC3488272

[20] Mentzel T, Brown LF, Dvorak HF The association between tumour progression and vascularity in myxofibrosarcoma and myxoid/round cell liposarcoma. Virchows Arch. 438(1), 13–22 (2001). 11213831 10.1007/s004280000327

[21] Nozawa H, Chiu C, Hanahan D. Infiltrating neutrophils mediate the initial angiogenic switch in a mouse model of multistage carcinogenesis. Proc. Natl Acad. Sci. USA 103(33), 12493–12498 (2006). 16891410 10.1073/pnas.0601807103 PMC1531646

[22] Farhood B, Najafi M, Mortezaee K. CD8^+^ cytotoxic T lymphocytes in cancer immunotherapy: a review. J. Cell. Physiol. 234(6), 8509–8521 (2019). 30520029 10.1002/jcp.27782

[23] Stone RL, Nick AM, McNeish IA Paraneoplastic thrombocytosis in ovarian cancer. N. Engl. J. Med. 366(7), 610–618 (2012). 22335738 10.1056/NEJMoa1110352 PMC3296780

[24] Bremnes RM, Busund LT, Kilvaer TL The role of tumor-infiltrating lymphocytes in development, progression, and prognosis of non-small cell lung cancer. J. Thorac. Oncol. 11(6), 789–800 (2016). 26845192 10.1016/j.jtho.2016.01.015

[25] Movahedi K, Laoui D, Gysemans C Different tumor microenvironments contain functionally distinct subsets of macrophages derived from Ly6C(high) monocytes. Cancer Res. 70(14), 5728–5739 (2010). 20570887 10.1158/0008-5472.CAN-09-4672

[26] Qian BZ, Pollard JW. Macrophage diversity enhances tumor progression and metastasis. Cell 141(1), 39–51 (2010). 20371344 10.1016/j.cell.2010.03.014 PMC4994190

[27] Miyagawa Y, Yanai A, Yanagawa T Baseline neutrophil-to-lymphocyte ratio and C-reactive protein predict efficacy of treatment with bevacizumab plus paclitaxel for locally advanced or metastatic breast cancer. Oncotarget 11(1), 86–98 (2020). 32002126 10.18632/oncotarget.27423 PMC6967770

[28] Passardi A, Scarpi E, Cavanna L Inflammatory indexes as predictors of prognosis and bevacizumab efficacy in patients with metastatic colorectal cancer. Oncotarget 7(22), 33210–33219 (2016). 27120807 10.18632/oncotarget.8901 PMC5078087

[29] Trinh H, Dzul SP, Hyder J Prognostic value of changes in neutrophil-to-lymphocyte ratio (NLR), platelet-to-lymphocyte ratio (PLR) and lymphocyte-to-monocyte ratio (LMR) for patients with cervical cancer undergoing definitive chemoradiotherapy (dCRT). Clin. Chim. Acta 510, 711–716 (2020). 32919942 10.1016/j.cca.2020.09.008

[30] Salati M, Filippi R, Vivaldi C The prognostic nutritional index predicts survival and response to first-line chemotherapy in advanced biliary cancer. Liver Int. 40(3), 704–711 (2020). 31773848 10.1111/liv.14314

[31] Zhang J, Zhang HY, Li J, Shao XY, Zhang CX. The elevated NLR, PLR and PLT may predict the prognosis of patients with colorectal cancer: a systematic review and meta-analysis. Oncotarget 8(40), 68837–68846 (2017). 28978160 10.18632/oncotarget.18575 PMC5620300

[32] Mandaliya H, Jones M, Oldmeadow C, Nordman II. Prognostic biomarkers in stage IV non-small cell lung cancer (NSCLC): neutrophil to lymphocyte ratio (NLR), lymphocyte to monocyte ratio (LMR), platelet to lymphocyte ratio (PLR) and advanced lung cancer inflammation index (ALI). Transl. Lung Cancer Res. 8(6), 886–894 (2019). 32010567 10.21037/tlcr.2019.11.16 PMC6976360

[33] Miyamoto R, Inagawa S, Sano N, Tadano S, Adachi S, Yamamoto M. The neutrophil-to-lymphocyte ratio (NLR) predicts short-term and long-term outcomes in gastric cancer patients. Eur. J. Surg. Oncol. 44(5), 607–612 (2018). 29478743 10.1016/j.ejso.2018.02.003

[34] Liu ZL, Zeng TT, Zhou XJ Neutrophil–lymphocyte ratio as a prognostic marker for chemotherapy in advanced lung cancer. Int. J. Biol. Markers 31(4), e395–e401 (2016). 27416842 10.5301/jbm.5000222

[35] Chan SWS, Smith E, Aggarwal R Systemic inflammatory markers of survival in epidermal growth factor-mutated non-small-cell lung cancer: single-institution analysis, systematic review, and meta-analysis. Clin. Lung Cancer 22(5), 390–407 (2021). 33582072 10.1016/j.cllc.2021.01.002

[36] Cao D, Xu H, Xu X, Guo T, Ge W. A reliable and feasible way to predict the benefits of nivolumab in patients with non-small cell lung cancer: a pooled analysis of 14 retrospective studies. Oncoimmunology 7(11), e1507262 (2018). 30377569 10.1080/2162402X.2018.1507262 PMC6205035

[37] Li B, Wang S, Li C The kinetic changes of systemic inflammatory factors during bevacizumab treatment and its prognostic role in advanced non-small cell lung cancer patients. J. Cancer 10(21), 5082–5089 (2019). 31602260 10.7150/jca.30478 PMC6775608

[38] Zikos TA, Donnenberg AD, Landreneau RJ, Luketich JD, Donnenberg VS. Lung T-cell subset composition at the time of surgical resection is a prognostic indicator in non-small cell lung cancer. Cancer Immunol. Immunother. 60(6), 819–827 (2011). 21373990 10.1007/s00262-011-0996-4 PMC4154502

